# Experience of a Northern California Acute Care Hospital in Active Surveillance for Candida auris

**DOI:** 10.1017/ash.2025.417

**Published:** 2025-09-24

**Authors:** Eugenia Miranti, Humberto Martinez, Guillermo Rodriguez Nava, Jorge Salinas, Mindy Sampson, Gina Newman

**Affiliations:** 1 Stanford Health Care; 2 Stanford University School of Medicine; 3 Stanford University; 4 Stanford University; 5 Stanford Healthcare

## Abstract

**Background:** Candida auris is an emerging fungal pathogen with potential to cause outbreaks. To mitigate transmission, the California Department of Public Health (CDPH) recommends considering implementation of an active case detection process in acute care hospitals to identify high-risk patients who may be colonized on admission. **Methods:** From 1/5/2024 to 9/20/2024, Stanford Health Care piloted an active surveillance program to identify high-risk patients for C. auris – defined as patients coming from (1) long term acute care hospitals (LTACHs), (2) ventilator skilled nursing facilities (vSNFs), (3) outside institutions with known C. auris outbreaks, (4) hospitals in Nevada, (5) recent international hospitalizations, and (6) patients with carbapenemase-producing organism (CPO) colonization.

Patients were identified for screening via daily review of custom-designed lists from the electronic medical record (EMR). A list of patients admitted from skilled nursing facilities (SNFs) and a list of patients transferred to Stanford from an outside facility were cross-referenced with a published list of high-risk facilities provided by CDPH. A list of inpatients flagged for CPOs was reviewed daily. Infection prevention was also notified by the transfer center or the care team if a patient had a recent international hospitalization. Screening was via superficial skin specimens from the axilla and groin. Culture-based testing was performed with identification of any fungal growth via MALDI-TOF. **Results:** During the pilot period, 1159 patients were evaluated for high-risk criteria; 58 (5%) met criteria for C. auris testing. One of 58 patients (colonization (Figure 1). There were 5 clinical cases during the pilot period, including the patient identified via screening. Active surveillance required 5-7 hours per week of infection preventionist effort, plus an hour to educate nursing staff when screening tests were performed. **Conclusions:** Our experience with performing active surveillance for C. auris resulted in one positive case, suggesting that this approach may have a lower yield in regions with low prevalence. Since surveillance can often be a time-intensive task for infection preventionists and nursing staff, it is important we continue to improve our knowledge about when and what surveillance is the most effective.

